# Editorial: Disease-modifying approaches in type 1 diabetes

**DOI:** 10.3389/fendo.2025.1624146

**Published:** 2025-05-29

**Authors:** Benjamin Udoka Nwosu, Joseph M. Pappachan, Ambika P. Ashraf

**Affiliations:** ^1^ Donald and Barbara Zucker School of Medicine, Hofstra University, Hempstead, NY, United States; ^2^ Faculty of Science (Department), Manchester Metropolitan University, Manchester, United Kingdom; ^3^ University of Alabama at Birmingham, Birmingham, AL, United States

**Keywords:** type 1 diabetes mellitus, disease modifying agents, partial clinical remission, lipids, glucose

Type 1 diabetes (T1D) is a disorder that is marked by persistent hyperglycemia due to autoimmune destruction of pancreatic β-cells, necessitating lifelong dependence on exogenous insulin for survival. TID places a heavy burden on the individual and the national healthcare budgets because of its short- and long-term complications, such as severe hypoglycemia, diabetic ketoacidosis, blindness, diabetic neuropathy, amputations, kidney failure, and atherosclerotic cardiovascular disease. As a result, there have been calls for disease-modifying therapies that can target the root cause of T1D, preserve endogenous insulin production, and ultimately alter the trajectory of T1D and reduce complications. Initial efforts have centered on immunosuppression and immunotherapy. However, the failure of these approaches to completely protect the β-cells has led to more critical thinking in the field.

This Research Topic aimed to capture these new ideas in the field. Reports for this Research Topic range from interventions to prolong the partial clinical remission (PR) phase of T1D, new theoretical frameworks such as the use of high-dose gamma-aminobutyric acid (GABA) molecule to prolong PR, generation of insulin-secreting cell lines using CRISPR technology, and the exploration of new lipid-based pathways via the theory of hyperlipidemic memory for disease modification. These innovative works will shape the future of diabetology. O’Donovan et al., kicked off the Research Topic by providing a robust review of the current disease-modifying therapies in T1D. Teplizumab is the first FDA-approved therapy to delay T1D, but it only postpones disease progression by a median of about 2 years in high-risk individuals. They suggested that prolonging residual β-cell function (RBCF) by these therapies could increase life expectancy by up to 14 years in children diagnosed with T1D at a young age. An original research article from Li et al. reported on developing engineered cells that can secrete endogenous insulin as a promising therapeutic approach to T1D, evading autoimmune attacks, and reducing reliance on exogenous insulin administration. Using CRISPR/Cas9 gene editing and homology-directed repair, they precisely integrated a promoter-free EMCVIRES-insulin cassette into the 3’ untranslated region of the *GAPDH* gene in human HEK-293T cells. The investigators demonstrated in mouse studies that the subsequent Cytopore 1 microcarriers are biocompatible and promote the long-term survival of insulin-producing cells *in vivo.* By inserting the insulin gene into a housekeeping gene locus without using an external promoter, the insulin can be expressed constitutively along with an essential gene, reducing the risk of silencing and ensuring stable insulin production. These non-endocrine cells secreted functional insulin and reduced hyperglycemia. This promoter-free genetic engineering strategy for insulin secretion and efficient cell transplantation could enhance disease-modifying therapeutic approaches in T1D.

The failure of immunosuppressants and immunomodulators to completely protect the β-cells has led to a newer focus on augmenting intrinsic β-cell health versus protection from autoimmune attacks to ensure prolonged RBCF. In this regard, Jing et al. propose oral adjunctive therapies that focus on β-cell health as candidates of interest for disease modification in T1D. They reviewed agents that target thioredoxin-interacting protein (TXNIP), especially TIX100, an oral antidiabetic drug that inhibits TXNIP. Verapamil, a calcium channel blocker, was previously shown to improve β-cell survival by suppressing TXNIP; TIX100 is a next-generation compound designed for this pathway. However, compared to verapamil, TIX100 has a reduced side effect profile, higher specificity, potency, and effectiveness, and reduces hyperglucagonemia and hepatic fat. By improving β-cell health without immunosuppression, a TXNIP inhibitor like TIX100 could potentially be repurposed to preserve β-cells in T1D, although it has so far been studied as an attractive agent for managing patients with type 2 diabetes. Along the lines of newer agents to promote intrinsic β-cell health and prolong PR, Mick and McCormick. explored the role of GABA molecule in patients with T1D regarding its known actions, such as the augmentation of pancreatic β-cell content, reduction of excess glucagon secretion, and the mitigation of T-cell-mediated immune destruction. They proposed that given the depletion of GABA in islets of patients with T1D, the repletion of GABA may have pharmacologic applications in these patients. This suggests that a threshold level of GABA might be necessary to exert therapeutic effects, potentially by more robustly activating GABA receptors on islet and immune cells. They made an important observation that high-dose GABA therapy would be more likely to elicit a positive metabolic outcome than regular supplementation in a similar approach to high-dose vitamin D supplementation to prolong PR in patients with T1D (1).

Given the rising prevalence of childhood obesity in children and adolescents with T1D, Resnick et al. recommended that glucagon-like peptide-1 receptor agonists (GLP-1RAs) be used to reduce the prevalence of obesity in patients withT1D and thus modify or blunt the trajectory of adiposity-driven cardiovascular complications. They reviewed the impact of insulin resistance (IR) in these patients and the practical steps to introduce GLP-1RAs in individuals with T1D. Addressing double diabetes (T1D with IR) with GLP-1RA class of drugs could also reduce hypoglycemia risk by markedly lowering total insulin requirement in such individuals. Along the same lines, Lei et al., reported in their meta-analysis on the safety and efficacy of Sotagliflozin, a dual inhibitor of sodium-dependent glucose transporter-1 and 2, in patients with T1D, that adjunctive Sotagliflozin could reduce the risk for cardiovascular disease, end-stage kidney disease, and fractures by improving metabolic profiles. However, it is important to note that SGLT inhibitors in T1D come with an increased risk of diabetic ketoacidosis; the meta-analysis suggests that with careful patient selection and monitoring, the benefits might outweigh the risks, suggesting a potential adjunctive role for Sotagliflozin in T1D management.

Mittal et al. focused on the paucity of data on the gene-environment interactions for the pathogenesis of T1D. They published an integrative perspective article aimed at characterizing gene-environment interactions in patients with T1D. They proposed using ‘omics’ (i.e., combine genomics, metabolomics, microbiome analysis, and exposomics) technology to determine the impact of environmental factors such as viruses, pesticides, gut dysbiosis, genetic, and epigenetic changes in triggering autoimmune response against pancreatic β-cells. They further called for investigations into ‘epidrugs’, which they described as agents that modify epigenetic changes, as novel therapies for T1D. Such epigenetic therapies (for example, DNA methylation or histone modification inhibitors) could potentially reprogram immune or β-cell gene expression profiles to a less auto-aggressive state. While this concept is in its infancy for T1D, the authors believe that targeting the epigenome could interrupt the disease process in ways traditional drugs have not done. They believe that this precision medicine approach could modify the trajectory of T1D and reduce the complications of the disease.

In a 23-year prospective, population-based, cohort study of 391 women with gestational diabetes mellitus (GDM), Luiro et al. showed that women with GDM who possessed 3 diabetes-associated autoantibodies in their first-trimester blood samples developed T1D within 7 years from the GDM pregnancy. They added that the progression to T1D was associated with a diagnosis of GDM at <30 years, lower BMI, and insulin requirements during GDM. This study suggests a trial of disease-modifying therapies for these women during their preclinical phase of T1D. In their view, Tandel et al. proposed that using multiplex antibody-detection-by-agglutination-PCR (ADAP) assay could be an ideal tool for T1D risk testing for large-scale stages 1 and 2 T1D testing in the general population. The ADAP technology allows highly sensitive and simultaneous detection of multiple autoantibodies with a minimal sample, which could make broad population screening for early T1D risk feasible. By identifying at-risk individuals (such as those with multiple autoantibodies) in the general population, one could intervene earlier with disease-modifying therapies.

Liu et al. reported that in Chinese adults with a 1-5-year history of T1D, RBCF was associated with higher time in range or near normoglycemia, suggesting that disease-modifying therapy could improve outcomes for these patients by prolonging their RBCF. Even a small amount of preserved endogenous insulin production can significantly stabilize blood glucose levels, reducing glycemic variability and dangerous extremes in glycemia. This underscores the clinical importance of therapies that preserve β-cell function: patients with preserved C-peptide tend to experience fewer hypoglycemic episodes and fewer complications, as seen in prior diabetes studies. Another publication by Xiong et al. reported on a predictive model for personalized postprandial glycemic response (PPGR) in Chinese patients with T1D, given the complexity of the Chinese diet compared to the Western diet. They found that the key predictors of PPGR were the premeal blood glucose level, blood glucose trend 30 minutes before a meal, and the carbohydrate-to-protein ratio of the meal. They recommended lower pre-prandial blood glucose and lower carbohydrate intake to maintain normal PPGR. Such a model could help tailor meal planning and insulin dosing for individuals, which is especially relevant as dietary patterns vary globally. By better predicting blood sugar excursions after meals, clinicians can personalize nutrition therapy in T1D, a strategy that, while not directly altering the autoimmune process, can mitigate marked glycemic variability and thereby reduce glucotoxicity or other metabolic stresses on the body.

Another study from China by Zhang et al. explored the dynamics of stimulated C-peptide concentrations and fasting and postprandial glucagon concentrations using a steamed bread meal tolerance test. They found that as T1D progresses, C-peptide levels decrease, and postprandial glucagon levels rise. They suggested that reducing postprandial hyperglucagonemia could be a disease-modifying therapy in T1D. In practice, this could mean developing treatments to suppress inappropriate glucagon release or action in T1D. For instance, adjunct therapies like GLP-1 agonists or glucagon receptor antagonists that specifically target α-cell activity. By curbing excessive glucagon release (which exacerbates hyperglycemia), one could improve overall glycemia and decrease the glucotoxic burden on surviving β-cells. In a review article, Nwosu expanded on his theory of hyperlipidemic memory of T1D, which explains the dichotomy in atherosclerotic cardiovascular (ASCVD) risk based on the presence or absence of PR. In this article, he proposes two fundamental ideas for disease-modifying therapies. The first is that any effort at complete β-cell protection must include lipid pathways to ensure a significant reduction in ASCVD risk. In other words, focusing only on glycemic control and autoimmunity is not sufficient; controlling dyslipidemia early in the course of T1D is crucial to prevent long-term cardiovascular complications. This idea arises from observations that some youth with T1D develop adverse lipid profiles very soon after diagnosis (especially if they did not experience a remission phase), which may set the stage for future cardiovascular disease. Secondly, PR is an imprimatur and not a process, suggesting that strategies to ensure the occurrence of PR in individuals with preclinical T1D will lead to more robust long-term outcomes than interventions to prolong the duration of PR following stage 3 T1D. This means that inducing a remission (even a short one) around the time of diagnosis or in the late preclinical phase might confer lasting metabolic benefits, perhaps by instilling a healthier metabolic memory, whereas trying to extend an established remission later may be less impactful. This concept challenges researchers to prioritize therapies that trigger remission in new-onset T1D (or prevent symptomatic onset altogether) as a strategy to imprint a lower-risk metabolic profile from the start.

In conclusion, this Research Topic provides a tour de force of the current strategies to protect the β-cells in T1D by reducing autoimmune attacks, augmenting intrinsic β-cell health, and exploring physiological, genetic, epigenetic, environmental, bioengineering, and population-based approaches to preserve β-cells, prolong RBCF, and reduce the medical and financial burdens of T1D around the world. The innovative concepts highlighted here will undoubtedly shape the future of diabetology and inspire further research into state-of-the-art disease-modifying therapies for T1D.
